# The Effect of Set Up Position on EMG Amplitude, Lumbar Spine Kinetics, and Total Force Output During Maximal Isometric Conventional-Stance Deadlifts

**DOI:** 10.3390/sports6030090

**Published:** 2018-08-31

**Authors:** Corey Edington, Cassandra Greening, Nick Kmet, Nadia Philipenko, Lindsay Purves, Jared Stevens, Joel Lanovaz, Scotty Butcher

**Affiliations:** 1 College of Kinesiology, University of Saskatchewan, Saskatoon, SK S7N 5B2, Canada; corey.edington@usask.ca (C.E.); joel.lanovaz@usask.ca (J.L.); 2 School of Rehabilitation Science, University of Saskatchewan, 104 Clinic Place, Saskatoon, SK S7N 0Z4, Canada; crg091@mail.usask.ca (C.G.); nik469@mail.usask.ca (N.K.); nyp897@mail.usask.ca (N.P.); lmp261@mail.usask.ca (L.P.); jared.stevens@usask.ca (J.S.)

**Keywords:** strength training, deadlift, biomechanics, lumbar spine, powerlifting, weightlifting

## Abstract

The purpose of this study was to examine the biomechanical differences between two set up variations during the isometric initiation of conventional barbell deadlifts (DL): Close-bar DL (CBDL), where the bar is positioned above the navicular, and far-bar DL (FBDL), where the bar is placed above the 3rd metatarsophalangeal joint. A cross-sectional, randomized, within-participant pilot study was used. Experienced powerlifters and weightlifters (*n* = 10) performed three individual isometric pulls of the initiation of both conditions. The CBDL resulted in lower tibia and knee angles and greater pelvis and torso angles than the FBDL (*p* < 0.05), as well as greater electromyography (EMG) activity in the biceps femoris and upper lumbar erector spinae, but lower activity in the vastus lateralis, and a lower knee extensor moment (*p* < 0.05). There were no statistical differences for ground reaction force, joint reaction lumbar shear and compression forces between the two conditions. Despite the differences in pelvis and torso angles between lifting conditions, the internal joint net moment, internal shear forces, and internal compressive forces were not different between the two lifting styles. The CBDL set up also resulted in greater posterior chain (hamstrings and erector spine) EMG amplitude, whereas the FBDL set up resulted in more anterior chain (quadriceps) amplitude. Lifters and coaches may choose either deadlift style, according to preferences or training goals, without concern for differences in lumbar spinal loading.

## 1. Introduction

The deadlift is a fundamental movement that has large translation to both everyday life and strength development and is a primary component in many strength training programs. An individual’s set up position of a deadlift is influenced by numerous anatomical parameters including hip and ankle range of motion, relative torso, arm, and leg length, and flexibility [1]. Consequently, many variations of the deadlift exist that may differentially accommodate specific anatomical and physiological characteristics, and to emphasize specific muscle groups [2]. For example, individuals who have reduced hip range of motion may opt for a conventional style over a sumo style because of the differences in set up position. [2] Choosing a deadlift style that complements a person’s anatomical characteristics can be advantageous in optimizing performance while decreasing injury risk, by allowing the lifter to maintain a relatively neutral spine and avoiding near maximal lumbar flexion [3]. 

Practically, there are two main variations of a conventional deadlift with different starting bar positions based on the barbell sports of powerlifting and weightlifting. In the powerlifting variation, the bar is placed directly above the navicular bone and below the inferior spine of the scapula, termed the close-bar deadlift (CBDL); in the weightlifting variation, the bar is placed above the metatarsophalangeal joint and below the acromioclavicular joint (AC), termed the far-bar deadlift (FBDL). As a result of the bar position, the CBDL style will have a higher hip position and more horizontal torso, which may result in increased net lumbar shear force and increased erector spinae activity. Hancock et al. [4], explored the differences in bar path due to each deadlift set-up position and found that the CBDL style results in 43% less horizontal bar displacement compared to the FBDL. It was suggested that the CBDL should give the trainee a more vertically linear line of pull during the lift, allowing them to lift heavier weight [4]. These different set up positions may have important implications for experienced lifters in their ability to generate force off the ground from the initiation of a lift.

Therefore, the purpose of this study was to compare the EMG amplitude, internal joint loads, and vertical isometric force output between the CBDL and FBDL set up positions using a maximal isometric deadlift initiation pull from the floor.

## 2. Materials and Methods

### 2.1. Research Design

A cross-sectional within-participant design was used for this pilot study. During a single testing session, participants performed the CBDL and the FBDL isometric pulls in a randomized order. The results of this pilot study will be used to design a larger trial of differences in set up positions during a full deadlift. Participants signed a consent form which was approved as part of the ethical approval granted by the University of Saskatchewan Biomedical Research Ethics Board (Bio: 16–82).

### 2.2. Participants

Ten participants (5 male, 5 female) were recruited for this study (age 32 years ± 10, height 172.48 cm ± 10.87, mass 88.73 kg ± 22.16, years of training experience 6.05 years ± 3.35). Table 1 contains additional demographic information for the participants. Participants were recruited who had trained for at least 2 years and completed at least one competition in either weightlifting (*n* = 4) or powerlifting (*n* = 6) at the Provincial or National level. While each participant had a dominant lifting style, all participants used both deadlift styles extensively as part of their training. Exclusion criteria consisted of individuals who had sustained a musculoskeletal injury within the past six months (defined as any tissue or joint damage that has caused the individual to either cease or seriously alter their regular training routine for longer than two weeks) and anyone with a diagnosed spinal deformity.

### 2.3. Procedure

Descriptive variables that were gathered from participants consisted of standing and sitting height, body mass, arm span, self-reported years of competition experience, and self-reported one repetition maximum. 

#### 2.3.1. Landmarking

Participants were landmarked for EMG electrode placement. EMG landmarks were based on placement suggested by Hamlyn et al. [5] and Seniam.org [6]. Physical therapy students completed landmarking of the foot, which consisted of palpating the barefoot to locate the navicular bone and third metatarsophalangeal joint (MTP). One sock was donned and the landmarked spots of the barefoot were used as a symmetrical guide for the socked foot. The same process of comparison and landmark transfer was performed over the sock of the remaining barefoot. These landmarks were used to standardize the two deadlift positions. Landmarking of the knees was achieved by palpation of the medial and lateral condyles of the femur. Landmarking of the spine was achieved by palpation and marking of the spinous processes. Palpation was verified by at least two researchers. 

#### 2.3.2. Warm-Up 

Subjects participated in a warm-up on a stationary bike (828E, Monark Exercise AB, Vansbro, Sweden) for 10 min. In order to avoid fatigue, the warm-up was performed unloaded at a comfortable pace and a heart rate monitor was used to ensure a low intensity was maintained (heart rate of less than 120 bpm). Participants were then provided the opportunity to perform any hip mobility exercises that would normally precede their weight training session. Although this was not standardized for each participant, the intent was to replicate their typical training methods in order to optimize their lifting technique. 

**Figure 1:**
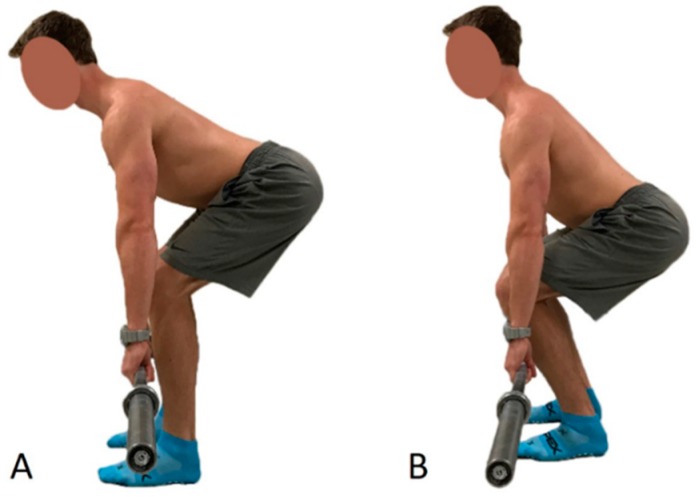
Demonstration of the (**A**) close bar deadlift and (**B**) far bar deadlift set up positions.

Standardized instructions were provided to the participants regarding warm-up and technique for the two deadlift variations. Both deadlift styles as well as a hip hinge were demonstrated to each participant and participants had the opportunity to practice three (or more as needed) isometric pulls in each position. Set up for each deadlift condition was as follows (as shown in Figure 1): the CBDL position (Figure 1A) consisted of the bar being placed directly over the navicular bone and below the inferior spine of the scapula. The FBDL (Figure 1B) consisted of the bar being placed directly over the third MTP joint and directly below the acromioclavicular joint. Following the practice lifts, participants performed 3 sets of 4 repetitions for each style of deadlift concentrically at 50, 60, and 70% of their self reported 1RM, respectively. Participants were asked not to lift greater than 70% of their self reported 1RM during warm-up to avoid fatigue due to the warm-up.

#### 2.3.3. Electromyography and Motion Capture Marker Placement 

Following warm-up, participants were immediately outfitted with EMG electrodes and motion capture markers. The EMG amplitude measured in this study were for biceps femoris, vastus lateralis, gluteus maximus, erector spinae (at the level of L1 and L5 vertebrae) and latissimus dorsi. The skin at the electrode sites was shaved and cleansed with an alcohol swab. All electrodes were placed on the participant’s dominant side. Biceps femoris electrodes were placed at 50% of the distance between the ischial tuberosity and the lateral epicondyle of the tibia. Vastus lateralis electrodes were placed two-thirds of the distance between the anterior superior iliac spine to the lateral aspect of the patella [6]. Gluteus maximus electrodes were placed at 50% of the distance between the S1 vertebrae and the greater trochanter [6]. Upper erector spinae electrodes were placed 6cm lateral to L1 and lower erector spinae electrodes 2cm lateral to L5 [5]. Lastly, latissimus dorsi electrodes were placed 1 cm lateral from inferior border of scapula, with the participant in standing, arms resting at sides [6]. 

Individual reflective markers were placed over the second metatarsophalangeal joints, acromioclavicular joints, elbow joints, ulnar styloid processes, posterior inferior iliac spines, calcanei, center of forehead and superior to the ears, as well as the following spinous processes: T1, T7, T10, L1, L3, L5. Additional markers for system calibration include: anterior superior iliac spines, medial and lateral malleoli and medial and lateral femoral condyles. These were used to obtain resulting moments at targeted joints.

#### 2.3.4. Equipment for Data Collection

Kinematic data was collected for all trials using an 8 camera VICON 3D motion capture system (version 2.1.1, VICON, Culver City, CA, USA) at a sampling rate of 100 Hz. This data was then processed using a 4th order Butterworth digital filter and NEXUS software (version 2.3, VICON, Culver City, CA, USA). The 3D marker trajectories were low-pass filtered at a cut-off frequency of 8 Hz. Clustered reflective markers were placed over the mid-tibia and mid-femur bilaterally, and sacrum. A telemetered electromyography (EMG) (Telemyo 2400T G2, Noraxon, Scottsdale, AZ, USA) device with surface electrodes was used to measure EMG amplitude. Six pairs of Neuroplus Ag/Ag–Cl rectangular (2.54 cm^2^) surface electrodes (Vermed, Bellows Falls, VT, USA; A10043) were placed over the bellies of each muscle in the direction of the line of action (20 mm inter-electrode distance). EMG data were sampled at 2000 Hz. Raw EMG data were high-pass filtered with a 20 Hz cutoff, full-wave rectified and then low-pass filtered at 100 Hz to generate a linear envelope.

Total ground reaction force (GRF) during the maximum isometric pull was collected using two in-ground force plates (OR6–7, AMTI, Watertown, MA, USA), one beneath each foot, at a sampling rate of 2000 Hz. All GRFs were low-pass filtered at a cut-off frequency of 250 Hz.

A barbell was weighted well beyond the lifter’s estimated 1RM to ensure an isometric pull. The bar was secured to the floor in order to prevent displacement during the isometric lift and was set at a height corresponding to a standard weightlifting plate (approximately 22.4 cm from the floor). The first starting position was randomized. Each participant performed three trials in both starting positions. Each trial consisted of a maximal isometric pull lasting three seconds and was followed by a two minute rest period to control for fatigue. Initial foot placement for each style was marked on the force plates so that each lift in its respective style was the same distance from the bar for each trial. GRF data were used to identify the active pulling time and the middle 1 second of data were averaged for analysis for each trial. The mean of the three trials was used for the data analysis.

### 2.4. Data Analysis

The virtual location of the L5-S1 joint was estimated from standing at the vertical level of the posterior superior iliac spine (PSIS) and anteriorly half-way between the PSIS and hip joint centres and tracked using pelvis markers. The kinematic and kinetic data were then used to calculate joint angles and subsequent moments at the knee, hip and L5-S1 joints using standard inverse dynamics techniques via custom routines written in Matlab (v2006b, Mathworks, Natick, MA, USA). Knee and hip joint moments were averaged across both limbs. Lumbar angle was measured by taking the relative angle between two vectors connecting the L5, L3, and L1 markers.

To estimate joint net shear and compression forces of each lift variation, a model was used such that the lumbar extensor muscles were represented by a single equivalent muscle (SEM) [7]. The SEM was assumed to have a moment arm with respect to the L5/S1 joint of 4 cm and an angle of pull of 10 degrees. The SEM values were estimated using the model presented by van Dieen et al. [8] with the lumbar angle set to its maximum flexion. The net lumbar moment and reaction forces were combined with the SEM to estimate compression and shear at L5/S1.

### 2.5. Statistical Analyses

Participant characteristics were analysed using descriptive statistics. Data was checked for normalcy and was normally distributed. Paired t-tests were utilized to detect significant differences between the CBDL and FBDL position variables as described above. Alpha was set a priori to 0.05. All statistics were analysed using Statistica 8.0 (StatSoft, Tulsa, OK, USA).

## 3. Results

Upon analysis of the data, two participant’s results were shown to be inaccurate due to technical issues rendering their data unsuitable, and therefore were not included in the results. Thus, data for *n* = 8 participants were included in the statistical analysis.

### 3.1. Angles

The reflective markers were used to determine the angle of the foot, tibia, knee, pelvis and torso for both deadlift postures during the pull. The joint angle results for each participant and the mean and standard deviations can be found in Table 2. A significant difference between the two deadlift positions was found for the angle of the tibia, the knee, the pelvis, and the torso (*p* < 0.05). 

### 3.2. Electromyography

When comparing the EMG amplitude between the FBDL and CBDL set-ups, there was a significant difference observed for the upper lumbar erector spinae, biceps femoris and vastus lateralis (*p* < 0.05). Specifically, the FBDL produced greater EMG amplitude in the vastus lateralis in comparison to CBDL, while the CBDL had greater amplitude in the erector spinae and biceps femoris. Participant’s specific EMG amplitude values and means are reported in Table 3.

### 3.3. Moments

See Table 4 for individual joint extensor moments of each participant and the mean values per condition. A significant difference of 0.002 (*p* < 0.05) was found at the knee, with the FBDL producing a larger moment than the CBDL. No significant differences were found for the hip or L5-S1 joints.

### 3.4. Ground Reaction Force

There were no significant differences between the mean total GRF and the mean horizontal GRF. See Table 5 for individual and mean GRF data.

### 3.5. Lumbar Shear and Compression Force

Table 5 also shows participant lumbar shear and compression forces. There were no significant differences between the FBDL and CBDL for both shear and compression.

## 4. Discussion

We studied the kinematic, kinetic, and EMG amplitude differences between the Powerlifting style CBDL and the Weightlifting style FBDL set ups during an isometric pull from the ground. This isometric pull in the two different positions represents an isometric initiation of the two set up techniques. We found that the CBDL results in significantly less tibia and knee angles, but greater pelvis and torso angle. These findings represent the typically higher hip and greater torso lean observed with conventional Powerlifting style deadlifts. As expected, then, the EMG amplitude observed corresponded with the differences in set up position, whereby the CBDL had greater bicep femoris and upper lumbar erector spinae activity (components of what is also known as the “posterior chain”) and lower vastus lateralis activity in combination with lower knee extensor moment (components of the “anterior chain”). Despite these kinematic and EMG differences; however, there were no differences between the two lifting conditions for total ground reaction force, nor for joint reaction shear and compression forces.

The amount of torque in a deadlift is dependent on the distance of the center of mass of the upper body and the load from the point of rotation, which is the hips [9]. Consequently, the moment arm and subsequent torque developed can be increased with either a more horizontal trunk position or by increasing the distance of the bar from the axis of rotation. Shear force occurs when force is acting tangential to its longitudinal axis [9]. A component of the torque creates shear force in the lumbar spine, however, this torque may be applied due to the external load (force applied to the bar plus bodyweight) or to the internal joint reaction force, which likely has greater implications for tissue load, adaptation, and injury. It is often assumed that the shear created by the external load and the more horizontal torso posture of the CBDL set up results in greater lumbar shear forces, but our results using the internal joint reaction forces suggest that this is a false assumption.

Swinton et al. [10] found that a more vertical torso achieved due to the use of a hexagonal bar deadlift (HBDL) decreases the moment and subsequent shear force on the lumbar spine, however, shear in this case, was measured externally. Potvin et al. [9] demonstrated that increased activation in the erector spinae musculature serves to decrease shear force in the lumbar spine by counteracting the force of the external load in squatting movements. However, these findings are dependent on the ability to avoid a flexed posture (i.e., maintain ‘neutral’) when lifting, as paraspinal muscle activity decreases in more extreme flexed postures. In addition to trunk angle, increased distance of the bar from the axis of rotation will increase torque. Thus, positions that increase horizontal distance of the bar to the centre of mass (i.e., FBDL) increase torque and external shear forces in the lumbar spine [8,11,12]. Despite this, it appears that activation of the lumbar erectors by maintaining a ‘neutral’ position is sufficient to counteract these forces [9]. This suggests that the avoiding extreme lumbar spine flexion is more important to controlling shear force in the spine than the starting bar position. This supports findings offered by Wallden et al. [13], in that significant movement toward a flexed spine during a deadlift will increase shear forces and suggests that it may contribute to low back injury or pain.

As a result of the altered torque and moments created by different deadlifting positions, recruitment patterns of specific muscle groups are also altered to counteract these rotational forces. Sumo style deadlift and HBDL both result in similar positional deviations from the CBDL as our FBDL experimental set up in that the torso is more vertical and the knees have more flexion. These two deadlift variations have been shown to increase the knee moment and torque in comparison to a conventional deadlift. This results in increased activation of the quadriceps to counteract this moment [8,12,14,15] as well as a reduction in the biceps femoris activation [15]. This was also demonstrated in the present study for the FBDL, as the increased knee extensor moment corresponded with increased quadricep activation and reduced biceps femoris activation. In addition, the changes in torso angle result in a difference in the torque placed on the low back [10]. The more horizontal torso position in CBDL increased torque on the spine, and consequently increased activation of the lumbar paraspinals to counteract this torque. This demonstrates that alterations in body and bar position can result in significant changes in EMG amplitude and biomechanical outcomes of a lift but have little impact on the internal joint moments and reaction forces.

Hancock et al. [4] suggested that the CBDL may be more efficient as it minimizes the horizontal displacement by 43% of the bar during the lift. This has important implications for experienced weightlifters, as a more vertical line of pull may increase their ability to lift greater loads while maintaining a neutral spine. Swinton et al. [10] measured efficiency of a HBDL compared to a conventional deadlift and found horizontal displacement was reduced by 75% in the HBDL, allowing the athletes to have a significantly greater 1RM. However, in our examination of an isometric pull, we found no significant difference between the FBDL or CBDL setups in terms of total GRF, specifically vertical GRF. In addition, the HBDL, which most closely resembles the FBDL in the current study, showed increased quadricep activation, which may contribute to greater force output [1,10]. While the FBDL increased quadricep activation in the current study, no change in vertical force output was seen. Branch et al. [16], demonstrated that an increase in knee extensor musculature activation with increased knee extensor moments may act more to stabilize the knee, rather than extend it, which would be consistent with the lack of increase in ground reaction force found in our study. Therefore, because force output does not differ between set-up conditions, any changes in lifting ability during a full deadlift are likely not due to the set-up position force output per se, but rather to the bar path efficiency found by Hancock et al. [4]. While shear and compression forces are highest in the deadlift at setup [17], the present study only examined forces at this point of the lift. This does not provide insight into differences that occur throughout a dynamic movement. Further research is needed to determine the total vertical force output, as well as internal joint reaction shear and compression forces between dynamic CBDL and FBDL setups.

It should be noted that the small sample size of this pilot study and the isometric nature of the efforts limit generalizability of these results. Larger trials utilizing a full deadlift are required to enhance the applicability of these findings. In addition, although all participants were experienced with both deadlift styles, it is possible that participants’ preference of a particular style may have influenced their performance and/or positioning.

## 5. Conclusions

A lack of literature currently exists regarding the biomechanical differences between different styles of conventional deadlifts. The present study examined the differences in biomechanical outcomes between two deadlifting styles in experienced athletes. The FBDL resulted in greater lumbar paraspinal and biceps femoris EMG, while the CBDL resulted in greater quadricep EMG forces. No differences existed between the two lifting styles in regards to internal shear and compression forces in the lumbar spine or total force output. These findings suggest that a lifter should use a deadlifting position that complements their anatomical characteristics and/or training goals, as shear force and performance are both maintained similarly during each position at the start of the lift.

### Practical Applications

Exercise selection may be a key determinant of resistance training program effectiveness. It affects the main adaptations that occur in response to training, and thus exercises chosen should function to target the desired muscle groups without compromising a trainee’s safety. The deadlift is a common exercise in resistance training programs as a whole-body strength exercise. However, little consideration is often given to variations in bar alignment and the effect it has on biomechanical outcomes. The present study demonstrates that aligning the bar over the navicular versus aligning the bar over the MTP does result in a significant change. The setup position of a deadlift influences movement mechanics and resultant EMG amplitude. Thus, if a specific training outcome or rehabilitation goal is targeted, the CBDL and FBDL setups should be differentiated and chosen accordingly. When the training goal is to target quadricep activation and decrease the workload on the erector spinae, the FBDL setup should be chosen. If the goal is to increase activation of the posterior chain including biceps femoris and erector spinae, the CBDL should be the setup chosen. In the instance that an athlete has no preference over muscle activation patterns and no specific rehabilitation goals, both the CBDL and FBDL are viable options because our study findings indicating no differences in lumbar internal shear and compression forces, as well as no differences in total ground reaction force.

## Figures and Tables

**Table 1:** Participant demographic data.

Characteristic	Mean (Standard Deviation)
Years of experience (years)	6.05 (3.35)
Mass (kg)	88.73 (22.16)
Body Mass Index (kg/m^2^)	29.73 (7.00)
Standing height (cm)	172.48 (10.87)
Arm length (cm)	52.34 (3.57)
Femur length (cm)	38.42 (4.22)
Tibia length (cm)	40.99 (3.06)
Torso length (cm)	42.67 (2.90)
Reported deadlift 1RM (kg)	179.85 (63.06)

1RM = 1 repetition maximum.

**Table 2:** Individual and Mean Joint Angles.

#	Foot Angle (°)		Tibia Angle (°)		Knee Angle (°)		Hip Angle (°)		Pelvis Angle (°)		Lumbar Angle (°)		Torso Angle (°)	
	FBDL	CBDL	FBDL	CBDL	FBDL	CBDL	FBDL	CBDL	FBDL	CBDL	FBDL	CBDL	FBDL	CBDL
2	15.5	12.10	22.26	8.16	64.36	47.50	87.69	90.42	45.71	50.87	11.23	12.17	71.16	78.88
3	9.34	8.30	20.52	7.11	60.40	43.22	71.28	70.21	29.72	33.52	24.42	25.23	72.37	77.57
5	11.38	6.51	27.02	12.48	81.96	57.80	67.10	67.48	12.46	21.98	25.07	27.31	65.15	80.32
6	16.52	13.13	23.76	12.89	72.77	55.53	61.00	58.13	11.71	15.25	22.27	24.22	70.78	76.64
8	5.04	3.71	15.12	5.01	65.40	39.67	87.65	87.07	34.06	46.31	38.68	40.98	72.10	85.57
9	12.89	15.23	12.30	6.81	71.28	58.55	90.07	89.20	31.04	37.31	6.91	7.72	61.28	68.80
10	12.49	11.52	13.50	8.35	66.40	62.65	99.13	105.66	45.65	49.06	22.10	23.83	65.73	71.79
11	13.82	13.63	21.84	7.70	66.43	45.42	63.48	60.46	18.17	21.41	37.26	38.65	74.05	80.90
Mean ± SD	12.12 ± 3.64	10.52 ± 3.96	19.54 ± 5.29	8.56 * ± 2.75	68.63 ± 6.63	51.29 * ± 8.38	78.4 ± 14.35	78.70 ± 16.83	28.57 ± 13.49	34.36 * ± 13.77	23.49 ± 11.03	25.01 ± 11.42	69.0 ± 4.46	77.56 * ± 5.29

* significant difference from corresponding value for the FBDL set up position (*p* < 0.05).

**Table 3:** Individual and Mean EMG Amplitude.

#	Thoracic Erector Spinae (V)		Latissimus Dorsi (V)		Lower Lumbar Erector Spinae (V)		Upper Lumbar Erector Spinae (V)		Gluteus Maximus (V)		Biceps Femoris (V)		Vastus Lateralis (V)	
	FBDL	CBDL	FBDL	CBDL	FBDL	CBDL	FBDL	CBDL	FBDL	CBDL	FBDL	CBDL	FBDL	CBDL
2	2.40 × 10^−1^	2.82 × 10^−1^	1.00 × 10^−1^	1.21 × 10^−1^	1.14 × 10^−1^	1.26 × 10^−1^	1.42 × 10^−1^	1.45 × 10^−1^	2.11 × 10^−2^	2.18 × 10^−2^	5.22 × 10^−2^	8.09 × 10^−2^	4.95 × 10^−2^	2.77 × 10^−2^
3	5.80 × 10^−2^	5.40 × 10^−2^	2.93 × 10^−2^	2.70 × 10^−2^	5.33 × 10^−2^	6.57 × 10^−2^	1.06 × 10^−1^	1.22 × 10^−1^	5.86 × 10^−3^	4.83 × 10^−3^	1.68 × 10^−2^	2.95 × 10^−2^	4.61 × 10^−2^	2.79 × 10^−2^
5	7.79 × 10^−2^	1.01 × 10^−1^	2.57 × 10^−2^	5.34 × 10^−2^	7.82 × 10^−2^	6.92 × 10^−2^	1.06 × 10^−1^	1.34 × 10^−1^	2.40 × 10^−2^	1.54 × 10^−2^	1.40 × 10^−2^	2.60 × 10^−2^	7.20 × 10^−2^	4.56 × 10^−2^
6	9.98 × 10^−2^	1.07 × 10^−1^	1.16 × 10^−1^	2.77 × 10^−2^	3.99 × 10^−2^	3.96 × 10^−2^	1.13 × 10^−1^	1.32 × 10^−1^	1.57 × 10^−2^	1.56 × 10^−2^	2.62 × 10^−2^	4.12 × 10^−2^	4.47 × 10^−2^	4.38 × 10^−2^
8	1.02 × 10^−1^	9.54 × 10^−2^	2.42 × 10^−2^	2.15 × 10^−2^	8.86 × 10^−3^	7.97 × 10^−3^	3.45 × 10^−2^	3.46 × 10^−2^	1.74 × 10^−3^	1.95 × 10^−3^	1.34 × 10^−2^	7.49 × 10^−3^	9.91 × 10^−3^	8.01 × 10^−3^
9	9.18 × 10^−2^	9.89 × 10^−2^	1.79 × 10^−1^	1.94 × 10^−1^	66.14 × 10^−2^	6.59 × 10^−2^	1.09 × 10^−1^	1.18 × 10^−1^	1.62 × 10^−2^	1.69 × 10^−2^	1.86 × 10^−2^	3.48 × 10^−2^	4.48 × 10^−2^	4.13 × 10^−2^
10	9.43 × 10^−2^	8.74 × 10^−2^	1.45 × 10^−1^	1.28 × 10^−1^	4.59 × 10^−2^	4.72 × 10^−2^	1.24 × 10^−1^	1.26 × 10^−1^	2.10 × 10^−2^	1.45 × 10^−2^	2.58 × 10^−2^	2.53 × 10^−2^	5.31 × 10^−2^	4.19 × 10^−2^
11	1.49 × 10^−2^	1.89 × 10^−2^	1.07 × 10^−2^	1.15 × 10^−2^	3.40 × 10^−3^	4.49 × 10^−3^	1.04 × 10^−2^	1.20 × 10^−2^	1.60 × 10^−3^	2.02 × 10^−3^	3.97 × 10^−3^	4.51 × 10^−3^	7.09 × 10^−3^	6.21 × 10^−3^
Mean ± SD	9.73 × 10^−2^ ± 6.46 × 10^−2^	1.06 × 10^−1^ ± 7.73 × 10^−2^	7.88 × 10^−2^ ± 6.45 × 10^−2^	7.30 × 10^−2^ ± 6.67 × 10^−2^	5.06 × 10^−2^ ± 3.58 × 10^−2^	5.32 × 10^−2^ ± 3.87 × 10^−2^	9.30 × 10^−2^ ± 4.5 × 10^−2^	1.03 * × 10^−1^ ± 5.03 × 10^−2^	1.34 × 10^−2^ ± 9.07 × 10^−3^	1.16 × 10^−2^ ± 7.58 × 10^−3^	2.14 × 10^−2^ ± 1.44 × 10^−2^	3.12* × 10^−2^ ± 2.37 × 10^−2^	4.09 × 10^−2^ ± 2.19 × 10^−2^	3.03 * × 10^−2^ ± 1.49 × 10^−2^

* significant difference from corresponding value for the FBDL set up position (*p* < 0.05).

**Table 4:** Individual and Mean Joint Extensor Moments.

#	Knee Moment (N·m)		Hip Moment (N·m)		L5-S1 Moment (N·m)	
	FBDL	CBDL	FBDL	CBDL	FBDL	CBDL
2	36.40	0.73	282.62	288.36	683.03	670.74
3	31.58	8.17	131.72	141.35	422.20	413.23
5	313.63	85.66	206.37	239.30	621.85	682.57
6	86.89	48.29	264.81	282.90	779.92	837.33
8	49.11	6.93	177.25	171.42	507.91	463.69
9	1.89	13.53	184.81	189.69	398.45	415.36
10	8.09	13.94	202.60	190.56	391.01	344.87
11	59.15	25.81	148.91	169.29	472.70	528.18
Mean ± SD	50.59 ± 42.59	22.00 * ± 31.52	200.13 ± 52.68	209.12 ± 54.71	534.63 ± 144.50	544.50 ± 169.59

* significant difference from corresponding value for the FBDL set up position (*p* < 0.05).

**Table 5:** Individual and Mean Ground Reaction Force (GRF) and L5-SI Shear and Compression Forces.

#	Total GRF (N)		Horizontal GRF (N)		L5-S1 Shear Force (N)		L5-S1 Compression Force (N)	
	FBDL	CBDL	FBDL	CBDL	FBDL	CBDL	FBDL	CBDL
2	2702.79	2597.55	−28.75	−31.14	2965.16	2911.84	16,816.28	16,513.85
3	1590.88	1524.73	8.85	0.99	1832.86	1793.90	10,394.65	10,173.70
5	2068.55	2271.96	12.96	15.26	2699.56	2963.19	15,309.99	16,805.08
6	2525.44	2621.69	6.24	−4.00	3385.79	3635.03	19,201.76	20,615.28
8	1948.59	1904.42	−31.67	−44.04	2204.93	2012.96	12,504.79	11,416.06
9	1526.00	1557.32	41.06	10.29	1729.75	1803.15	9809.89	10,226.15
10	1516.33	1495.29	3.06	14.01	1697.45	1497.14	9626.71	8490.69
11	1951.34	2088.81	−22.90	23.19	20.52	2292.95	11,637.94	13,003.94
Mean ± SD	1978.74 ± 447.54	2007.71 ± 464.56	−2.16 ± 24.78	−7.73 ± 22.52	2363.77 ± 736.20	2320.95 ± 627.30	13,162.75 ±3357.57	13,405.59 ± 4175.22
